# A drug combination targeting hypoxia induced chemoresistance and stemness in glioma cells

**DOI:** 10.18632/oncotarget.24839

**Published:** 2018-04-06

**Authors:** Akansha Jalota, Mukesh Kumar, Bhudev C. Das, Ajay K. Yadav, Kunzang Chosdol, Subrata Sinha

**Affiliations:** ^1^ National Brain Research Centre, Manesar, Gurgaon-122051, India; ^2^ Department of Biochemistry, All India Institute of Medical Sciences, New Delhi-110029, India; ^3^ Dr. B. R. Ambedkar Center for Biomedical Research, University of Delhi, Delhi-110007, India; ^4^ Amity Institute of Molecular Medicine and Stem Cell Research, Amity University, Noida-201313, India

**Keywords:** BCNU, hypoxia, PGE2, chemosensitization, COX-2 inhibitor

## Abstract

Hypoxia is a characteristic of solid tumors especially Glioblastoma and is critical to chemoresistance. Cancer stem cells present in hypoxic niches are known to be a major cause of the progression, metastasis and relapse. We tried to identify synergistic combinations of drugs effective in both hypoxia and normoxia in tumor cells as well as in cancer stem cells. Since COX-2 is over-expressed in subset of glioblastoma and is also induced in hypoxia, we studied combinations of a prototype Cyclooxygenase (COX-2) inhibitor, NS-398 with various drugs (BCNU, Temozolomide, 2-Deoxy-D-glucose and Cisplatin) for their ability to abrogate chemoresistance under both severe hypoxia (0.2% O_2_) and normoxia (20% O_2_) in glioma cells. The only effective combination was of NS-398 and BCNU which showed a synergistic effect in both hypoxia and normoxia. This synergism was evident at sub-lethal doses for either of the single agent. The effectiveness of the combination resulted from increased pro- apoptotic and decreased anti-apoptotic molecules and increased caspase activity. PGE_2_ levels, a manifestation of COX-2 activity were increased during hypoxia, but were reduced by the combination during both hypoxia and normoxia. The combination reduced the levels of epithelial-mesenchymal transition (EMT) markers. It also resulted in a greater reduction of cell migration. While single drugs could reduce the number of gliomaspheres, the combination successfully abrogated their formation. The combination also resulted in a greater reduction of the cancer stem cell marker CD133. This combination could be a prototype of possible therapy in a tumor with a high degree of hypoxia like glioma.

## INTRODUCTION

WHO Grade IV glioma or Glioblastoma (GBM) are the most aggressive brain tumors. These are characterized by a hypoxic inner core which bestows the tumor with more aggressive properties including invasion, metastasis and chemoresistance. Hypoxia may induce drug resistance by the activation of several pathways as mediated by COX-2, PI3K pathway, AP1, c-Jun, Pim1 or Stat3 [1]. The hypoxic zone is known to harbor a subset of cells which have stem cell like properties and are known as cancer stem cells. These are characterized by alterations in genetic makeup, tumor initating, and differentiation capacity which are important for relapse of the disease [2–4]. Clinically used anti-tumor drugs (alkylating agents, metabolic inhibitors etc.) against glioma increase the medial survival of the patient only by several months. This may happen due to chemoresistance which can be attributed to the altered intrinsic and/or extrinsic (microenvironment/hypoxia related) factors. It is also well documented that chemotherapy gives rise to acquired chemoresistance (pan-resistance), in cancer cells as an adaptive mechanism [5]. A possible strategy to overcome the same is by using drug combinations with known activities. We have earlier shown the synergistic effect of 2-deoxy-D-glucose and cisplatin on cell monolayers under both hypoxia and normoxia. However this involved a distinct mechanism of conversion of autophagy to apoptosis, and also the effect on cancer stem cells was not demonstrated [6].

A well known mediator of pro-tumorigenic inflammation is COX-2. It is highly upregulated in many cancers including gliomas and is involved in tumor progression [7, 8]. COX-2 also helps in the proliferation of cancer stem cells [9]. It is responsible for the synthesis of prostanoids (prostaglandins, prostacyclin, and thromboxane) from the precursor arachidonic acid. Prostaglandins produced by COX-2 trigger the release of proinflammatory chemokines [10] . Indeed it is now well established that inflammation is a critical and enabling characteristic of tumorigenesis [11, 12]. This synergism towards tumor progression and metastasis makes this protein a potential therapeutic target. Since chemoresistance is very closely associated with hypoxia and COX-2 overexpression in tumor as well as stromal cells, inhibition of COX-2 activity may result in increased efficacy of conventional therapies (chemotherapy and radiation). In addition, there is also a possibility of COX-2 affecting angiogenesis, Epithelial Mesenchymal Transition (EMT) and spheroid formation which helps in tumor progression [13–16]. It has been reported by Tian *et al*. 2017 that TGF-β induced stemness in triple negative breast cancer is regulated by COX-2 [17]. Stemness property induced by COX-2 is also seen in colorectal cancer and its inhibitor in combination with EGFR inhibitor affect stemness related pathways [18].

Inhibiting COX-2 activity/expression suppresses the growth of various tumors [19, 20]. Several molecules (Etodolac, Celecoxib, NS-398 etc.) that specifically inhibit COX-2 expression/activity, reduce cell proliferation, angiogenesis, invasion and metastasis of tumor cells [21]. Several such COX-2 specific inhibitors are in preclinical or clinical trials though as of now their use is not very widespread [22–24]. NS-398 is a prototype of specific inhibitor of COX-2 that affects the activity of the enzyme. The inhibitory effect of this competitive inhibitor has been shown to result in reduced cell proliferation, invasiveness, metastasis, and increased apoptosis [7, 25]. However, NS-398 upregulates COX-2 expression at transcriptional level by a feedback loop mechanism [26], which may limit its efficacy. There are different reports on the effects of Celecoxib on EMT, the effect varies with the type of cancer and cell line. In colon cancer cells, celecoxib suppresses EMT [27]. However, celecoxib induces EMT in non-small cell lung carcinoma and epithelial ovarian cancer [28, 29] and this has been implicated in generation of a chemoresistant sub-population leading to tumor progression through invasion and metastasis [30, 31]. Since the heterogeneous populations within tumors need several altered pathways for their survival and progression, multi-modal drug combinations that affect different distinct pathways, may have a better therapeutic effect. Also, such combinations, especially involving COX-2 inhibitors may have better cytotoxicity at lower oxygen concentrations. Earlier studies showed that inhibition of COX-2 (by NS-398) along with epidermal growth factor receptor (EGFR) (through gefitinib) resulted in increased cytotoxicity of Docetaxel along with reduced cancer cell migration [32].

Hypoxia is known to induce cancer stem cells, which may result in increased therapeutic resistance for different modalities like chemotherapy and radiotherapy [2, 33–35]. Glioma stem cells (GSCs) are capable of tumor initiation, self renewal, and differentiation which leads to tumor heterogeneity, metastasis and repopulation of tumor after therapy [36] . Dedifferentiation of a tumor by hypoxia helps in the maintenance of stem like properties of tumor cells by the expression of genes like Oct4, SOX-2 and Nanog. CD133 is a known glioma cancer stem cell marker which helps in the identification of glioma cancer stem cells which show tumorigenic properties [37]. Hence there is a need to identify potential targets that regulate these glioma cancer stem cells in order to improve therapy. As COX-2 inhibitors are emerging as therapeutic agents for cancers, including cancer stem cells, it is important to study drug combinations that could improve their efficacy under both hypoxia and normoxia. Hypoxia also induces EMT which in turn is a regulator of stemness and hence a major contributor to drug resistance [38]. Therefore COX-2 inhibitors can be potential therapeutic agents for glioma cells. *In-vitro* studies of drug sensitivity and resistance are usually performed in 20% oxygen (atmospheric pressure). Even normal tissue under the best conditions of oxygenation rarely ever approaches this level of oxygenation. On the basis of EF5 binding technique which gives oxygen tension in the tissues as reported by Evan *et al* [39], WHO grade II tumors were characterized by modest cellular hypoxia (pO_2_~10%) and grade III tumors by modest-to-moderate hypoxia (pO_2_~10%–2.5%). Severe hypoxia, taken as approximately 0.1–0.2% O_2,_ was found in Grade IV tumors.

In this study we have exposed GBM cells under severe hypoxia (0.2% O_2_) and normoxia (20% O_2_) to various drug combinations in order to simulate the *in-vivo* tumor microenvironvement. NS-398 was taken as the prototype COX-2 inhibitor which was used in combination with the drugs (BCNU, Temozolomide (TMZ), Cisplatin (CP) and 2-Deoxy-D- glucose (2-DG)). While TMZ and BCNU are being used in glioma [40–42], CP and 2-DG have been tried earlier [6, 43–46]. We observed synergism under both hypoxia and normoxia, only with the combination of NS-398 and BCNU. This was reflected in the extent of decrease of the inflammatory modulator PGE_2_ which is the product of COX-2. We observed increased cell death with increased expression of pro-apoptotic markers. There was also decreased expression of the EMT markers and cell migration. Importantly the combination abrogated gliomaspheres formation and reduced CD133 expression.

## RESULTS

### Up regulation of COX-2 expression under hypoxia in glioma cells

**Figure 1:**
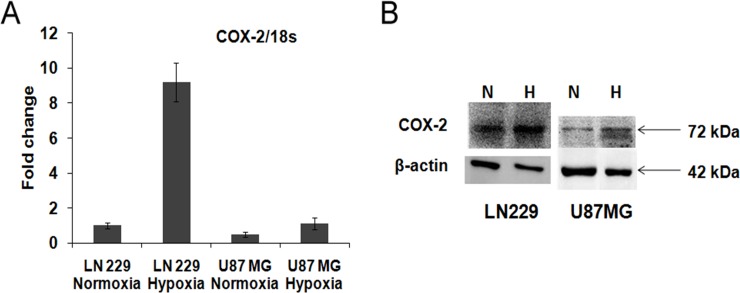
COX-2 expression in glioma cell lines (**A**) mRNA level expression. COX-2 expression was found in LN229 and U87MG cell lines and it was up-regulated under hypoxia in LN229 (9 fold change, *p* < 0.001) and U87MG cell line (2.2 fold change, *p* < 0.01). (**B**) Protein level expression. COX-2 protein expression was found in LN229 and U87MG cell lines and it was up-regulated under hypoxia in LN229 (1.9 fold change) and U87MG (1.3 fold change) cell lines. Lane N denotes Normoxia control, Lane H denotes Hypoxia control. β-actin was used as a control. Overall there is increase in both COX-2 mRNA and protein expression during hypoxia.

Effect of hypoxia on COX-2 expression was checked at the level of mRNA and protein in the glioma cell lines (U87MG and LN229), maintained under both hypoxic and normoxic conditions. We observed increased expression of COX-2 at both mRNA and protein level under hypoxia in both the cell lines (Figure 1). The expressions of hypoxia markers (CA9, VEGF and PGK1) as well as COX-2 mRNA were studied after exposure to severe hypoxia (0.2% O2) for 24, 48 and 72 hours. The hypoxia markers were upregulated at all the time points but the values at 48 and 72 hours were higher than at 24 hours in both the cell lines (Supplementary Figure 1(i)). COX-2 mRNA and protein expression were also increased after 24, 48 and 72 hours of hypoxia exposure in both the cell lines but the values at 48 and 72 hours were more than those for 24 hours. (Supplementary Figure 1(i, ii)).

### Dose dependent reduction in cell viability by the COX-2 inhibitor (NS-398) and BCNU in glioma cells under hypoxia and normoxia

**Figure 2:**
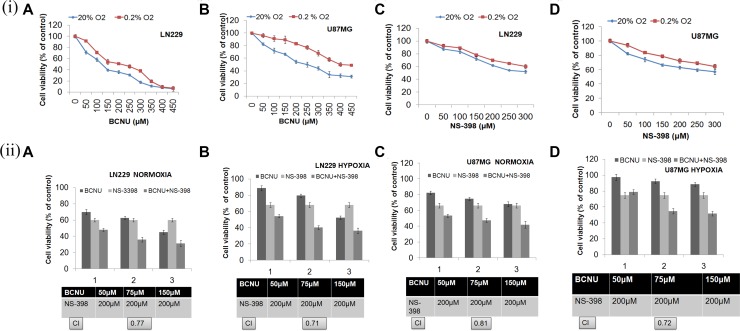
(**i**) Cell viability analysis at different doses of BCNU and NS-398. Both the cell lines were treated with increasing concentrations of BCNU and NS-398 and cell viability was assessed by MTT assay. Dose dependent cytotoxicity of BCNU and NS-398 was observed in (A, C) LN229 and (B, D) U87MG cell lines under hypoxia and normoxia. Results were expressed as mean ± SD of three experimental replicates. Under all conditions, drug effect of individual drugs was reduced under hypoxia. (**ii**) Cell viability analysis after treatment with the combination (BCNU + NS-398). Cells were treated with BCNU, NS-398 and BCNU + NS-398 and cell viability was assessed after 72 hours of treatment by MTT assay. The combination of BCNU + NS-398 (75 μM of BCNU and 200 μM of NS-398) showed synergism (CI < 0.9) under both hypoxia and normoxia in both cell lines: (A, B) LN229 and (C, D) U87MG cell lines. Results were expressed as mean ± SD of three experimental replicates. The combination had more effect than either single agent. This was true for normoxia (*p* < 0.01 with BCNU and *p* < 0.01 with NS-398) as well as hypoxia (*p* < 0.01 for BCNU and *p* < 0.01 for NS-398) for LN229 with reference to this synergistic combination. Also, for U87MG the levels of significance for normoxia were *p* < 0.01 for BCNU and *p* < 0.05 for NS-398. For hypoxia the levels of significance were *p* < 0.05 for BCNU and *p* < 0.05 for NS-398.

In order to study synergism with NS-398, firstly sub-lethal drug concentrations were determined by exposing cells to individual drugs - BCNU, CP, 2-DG and TMZ. Only sub-lethal doses were used to study the synergistic interaction of the combination (as calculated by Combination Index - CI). Of the several combinations tested, only the combination of NS-398 with BCNU showed synergistic effect (as determined by a CI of < 0.9) under hypoxia and normoxia in both the cell lines (Figure 2), (Supplementary Figure 2). This synergistic combination of BCNU and NS-398 at 75 μM of BCNU with 200 μM of NS-398, has been studied further.

### Increased apoptosis is induced by the combination of NS-398 and BCNU

**Figure 3:**
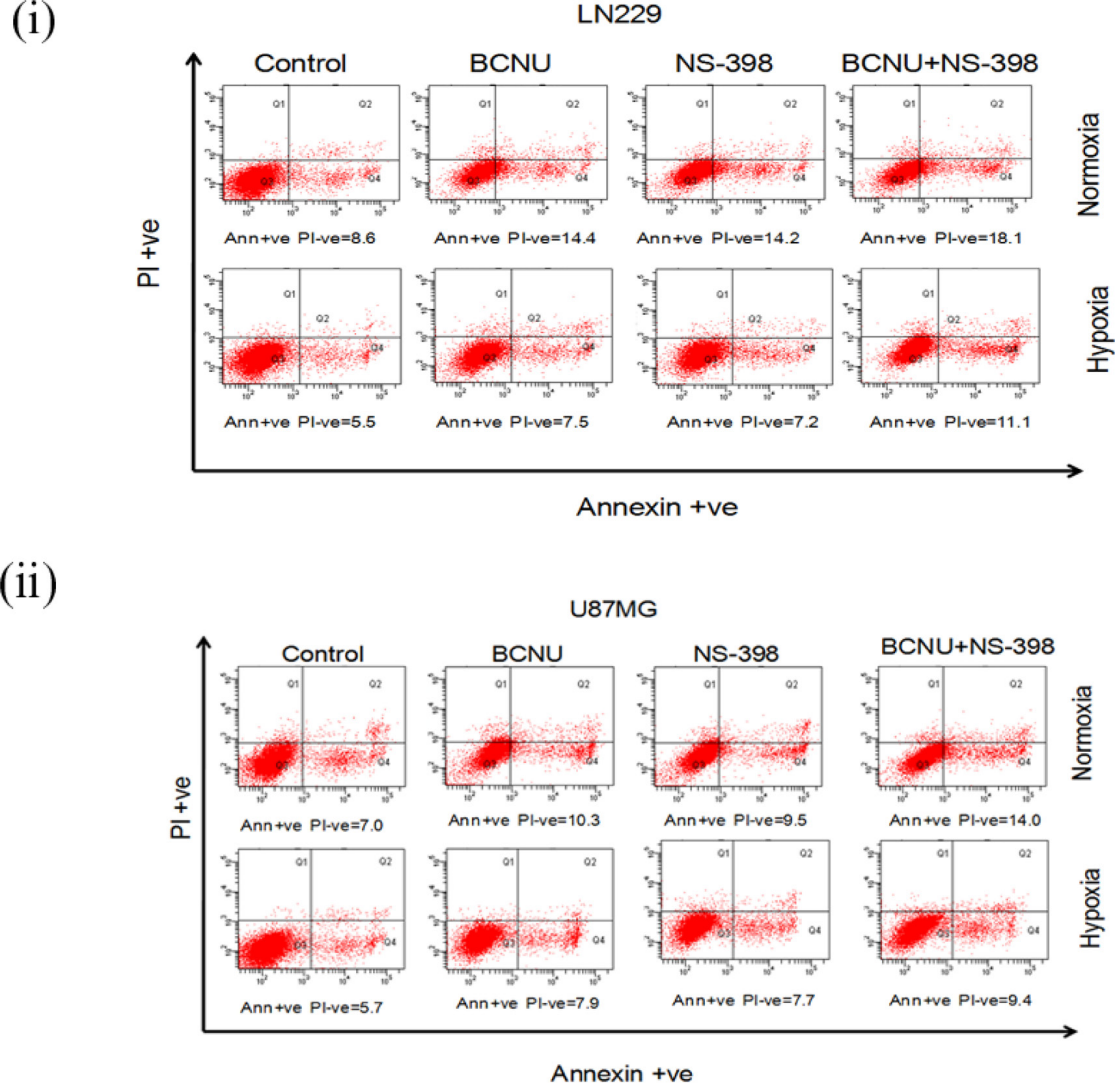
PI and Annexin V staining of glioma cells after drug treatment (**i**) LN229 and (**ii**) U87MG cells were treated with drugs (BCNU, NS-398 and combination) after 24 hours of normoxia and hypoxia and the same state continued throughout the experiment. PI and Annexin V staining was done after 48 hours of drug treatment. It was observed that there was a distinct increase in early apoptosis induced by the combination (BCNU + NS-398) as compared to single agent (BCNU or NS-398). Early apoptosis is depicted by Annexin V positive and PI negative cells. The percentage of cells with staining are depicted below each plot.

The mechanism of cell death by the combination was studied at the above mentioned concentrations of BCNU and NS-398 by propidium iodide staining (Supplementary Figure 3). Increased sub-G1 cells (indicating apoptosis) were observed by the combination under all the conditions tested (Supplementary Figure 3). This is in concordance with the reduction in cell viability by MTT assay. For apoptosis, we did a dual PI and Annexin V staining at 48 hours (to assess early apoptosis), and a PI staining at 72 hours for late apoptosis. At 48 hours, the dual PI and Annexin V staining revealed increased early apoptosis (increase in Annexin V positive and PI negative cells) for the combination as compared to the individual drugs (Figure 3(i, ii)). Correspondingly at 72 hours, the total apoptosis as revealed by increased sub G1 fraction in PI staining , was more in the combination (Supplementary Figure 3A and 3B represent LN229 and U87MG cell lines respectively).

### Apoptosis induced by the combination of BCNU and NS-398 is caspase dependent

**Figure 4:**
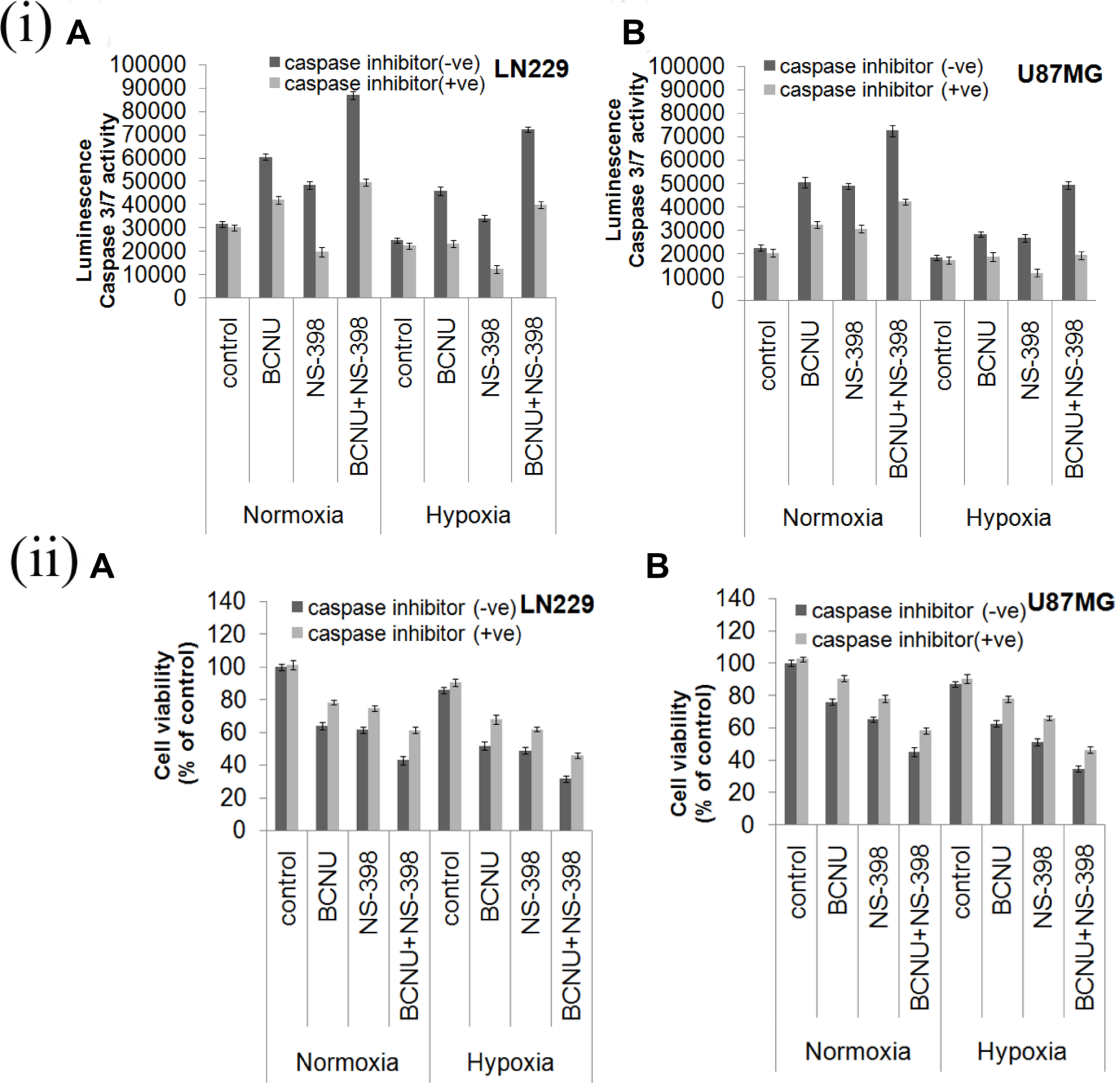
(**i**) Drug induced Caspase 3/7 activity and effect of caspase inhibitor. Cells were treated with BCNU, NS-398 and BCNU + NS-398 and caspase 3/7 activity was assessed after 48 hours. Caspase 3/7 activity was upregulated in the combination of BCNU and NS-398 as compared to single agents under both hypoxia and normoxia in (A) LN229 and (B) U87MG cell lines. Addition of caspase inhibitor reduced drug induced caspase activity. Results were expressed as mean ± SD of three experimental replicates. (**ii**) Cell viability analysis after treatment with caspase inhibitor. Both cells lines were treated with BCNU, NS-398 and BCNU + NS-398 in presence and absence of caspase inhibitor and MTT assay was done after 72 hours. The caspase inhibitor was able to reverse the decrease in cell viability by both single agent and combination in (A) LN229 and (B) U87MG cell lines. Overall the results indicate the role of caspase 3/7 activity in determining reduced cell viability, induced by the drug combination.

Caspase 3/7 activity was investigated to confirm whether the apoptosis induced by the combination was caspase dependent or independent. Caspase 3/7 activity (after 48 hours of drug treatment) was upregulated by the combination as compared to the single agent under all the conditions tested (Figure 4(i)). However the increase in the caspase 3/7 activity by the combination of BCNU and NS-398 was more in LN229 as compared to U87MG cell line under both the oxygen concentrations. Viability of both cell lines treated by the combination in presence and absence of inhibitor was assessed at 24, 48 and 72 hours Addition of the caspase inhibitor Q-VD-OPh (20 μM) to the individual drugs as well as to the combination of the drugs, resulted in a decrease in the caspase activity and increase in the cell viability (Figure 4(i), 4(ii)). The time kinetics of cell viability is depicted in Supplementary Figure 4. Cell death with the combination and its reversal with the inhibitor started at 48 hours and was more evident at 72 hours. This further confirmed that the apoptosis induced by the combination was caspase dependent.

### Apoptotic markers induced by the drug combination

**Figure 5:**
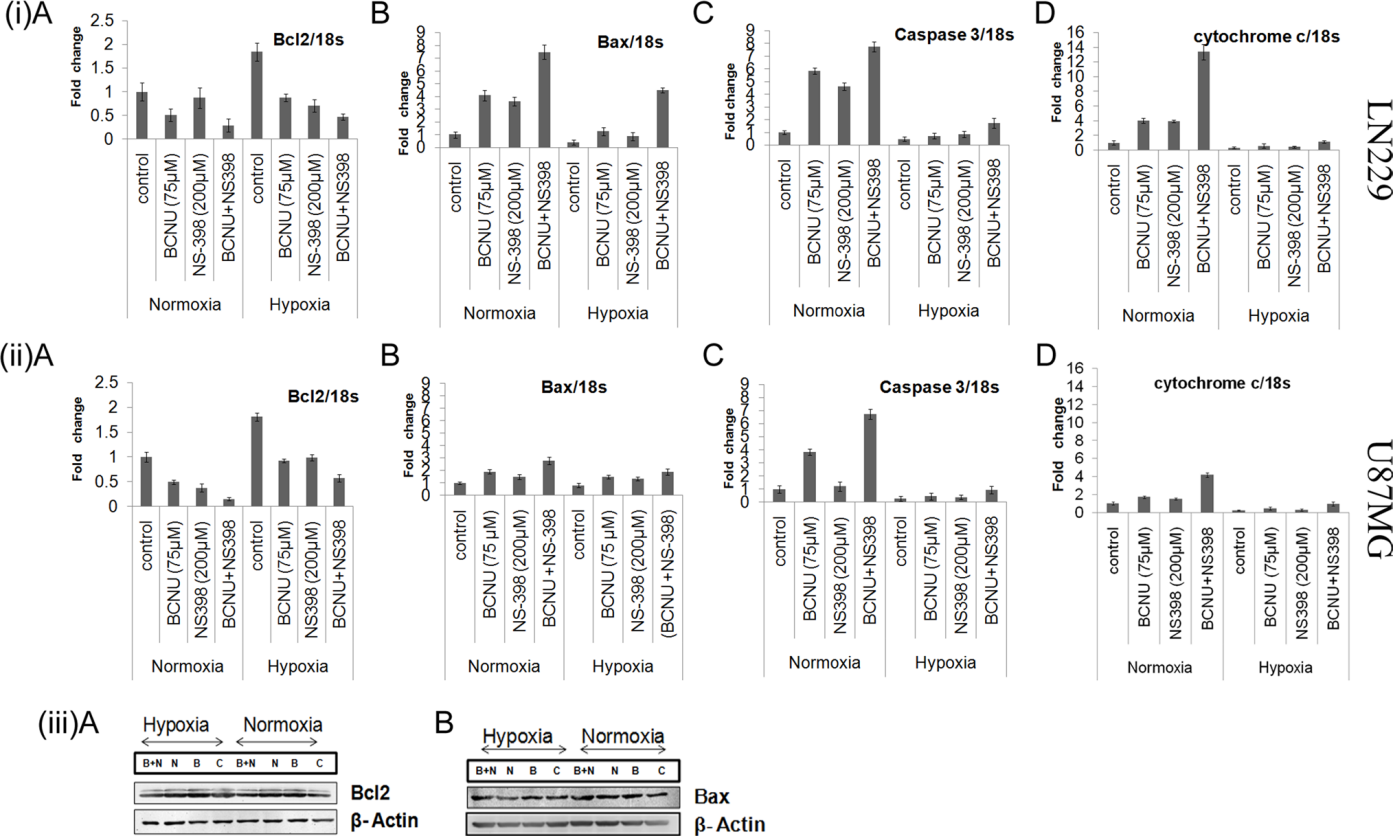
(**i**, **ii**) Expression of pro-apoptotic and anti-apoptotic markers under various conditions. This figure shows the quantitative mRNA expression for both pro-apoptotic markers (Bax, cytochrome c and caspase 3) and anti-apoptotic marker (Bcl2) by Real time PCR. There was more up-regulation of the pro-apoptotic markers and more down-regulation of the anti-apoptotic marker by the combination as compared to single agent under both hypoxia and normoxia in (i) LN229 and (ii) U87MG cell lines. Results were expressed as mean ± SD of three experimental replicates. (**iii**) Western blot analysis of Bcl2 and Bax in the LN229 cell line. The protein levels of Bcl2 and Bax were also studied in the LN229 cell line. Bax was up-regulated and Bcl2 was down-regulated by the combination as compared to single agent under normoxia and hypoxia. Lane C denotes control, Lane B denotes BCNU, Lane N denotes NS-398 and Lane (B + N) denotes BCNU + NS-398.

To further investigate the mechanism of increased apoptosis in the combination, pro-apoptotic markers (Bax, caspase 3, cytochrome c) and the anti-apoptotic marker (Bcl-2) were analyzed at the level of mRNA in the cells treated individually or by the combination. The expression of pro-apoptotic markers (Bax, caspase3 and cytochrome c) were found to be increased in both the cell lines treated with the combination as compared to the control or the single agents, under both hypoxia and normoxia. The degree of increase under normoxia was higher in the cells treated with the combination in comparison to hypoxia The increase in the levels of pro-apoptotic markers under hypoxia is however interesting in the context of their low base line. (Figure 5(i, ii)). Anti-apoptotic marker Bcl-2 was found to be reduced by the combination as compared to the control as well as by the single agent in both the cell lines under hypoxia as well as normoxia. However, the degree of reduction under normoxia was much more than hypoxia in both the cell lines treated with the combination. This may be due to the increased Bcl-2 expression level under hypoxia in both the cell lines (Figure 5(i, ii)). Further confirmation of the mRNA findings was done with protein analysis of representative pro (Bax) and anti-apoptotic (Bcl2) marker in the LN229 cell line (Figure 5(iii)). Bax was upregulated by the combination and Bcl2 was downregulated by the combination as compared to control and single agent.

### Reduced PGE_2_ activity induced by the drug combination under hypoxia and normoxia

**Figure 6:**
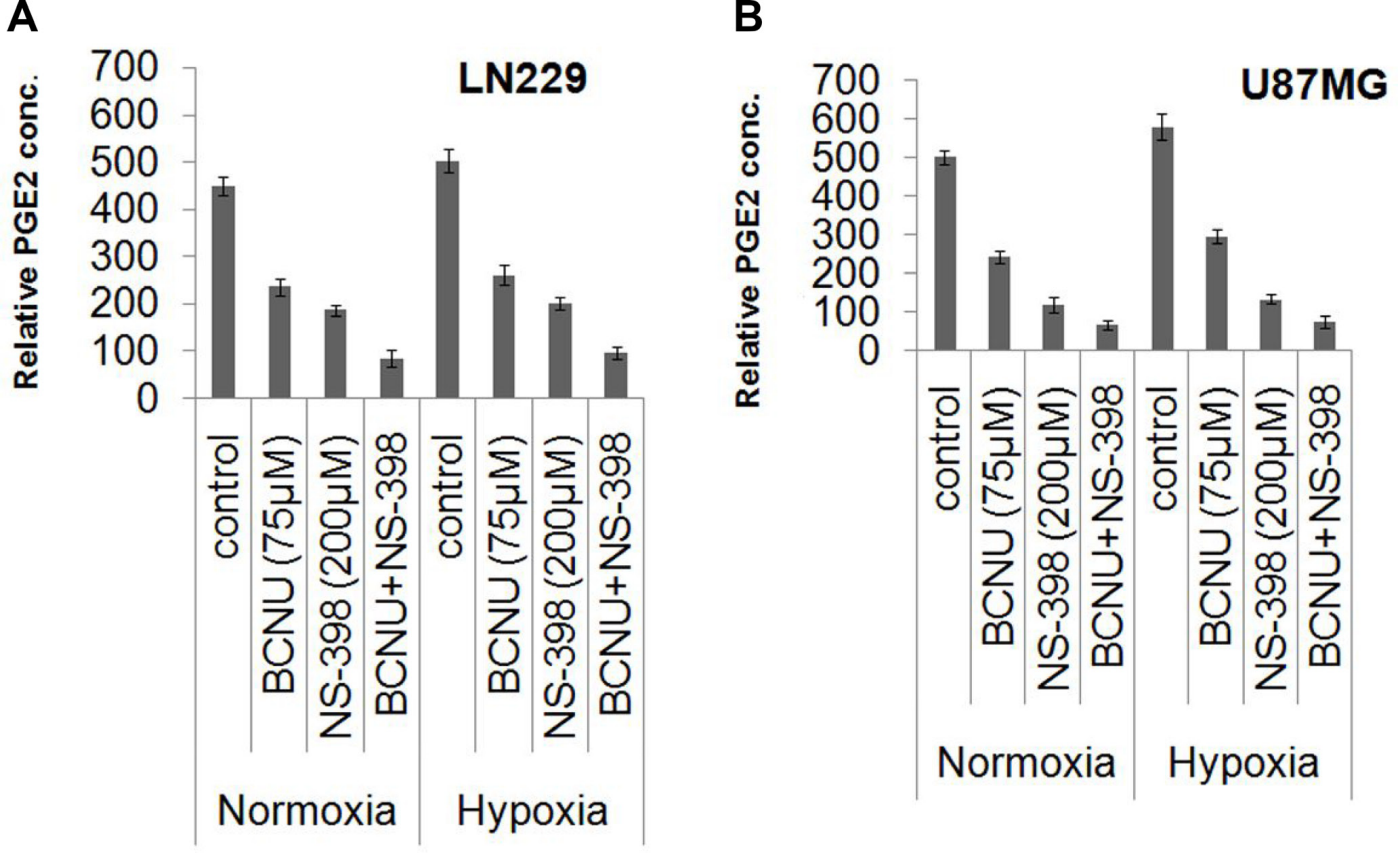
Relative PGE_2_ concentrations indicating COX-2 activity under various conditions Culture supernatants of drug treated (BCNU, NS-398 and BCNU + NS-398) and control samples of LN229 and U87MG cell lines under hypoxia and normoxia were assayed for PGE_2_ concentration by EIA kit. Relative PGE2 concentration was significantly reduced in the cells treated with the combination (BCNU + NS-398) under both hypoxia and normoxia in (**A**) LN229 and (**B**) U87MG cell lines. Results were expressed as mean ± SD of three experimental replicates. The combination had more effect than either single agent. This was true for normoxia (*p* < 0.01 with BCNU and *p* < 0.05 with NS-398) as well as hypoxia (*p* < 0.01 for BCNU and *p* < 0.01 for NS-398) for LN229. Also, for U87MG the levels of significance for normoxia were *p* < 0.05 for BCNU and *p* < 0.05 for NS-398. For hypoxia the levels of significance were *p* < 0.01 for BCNU and *p* < 0.05 for NS-398.

PGE2 levels were taken as a measure of COX-2 activity. These were reduced by both BCNU and NS-398 individually, and further lowered by the combination under hypoxia and normoxia in both the cell lines. Untreated hypoxic cells had a higher degree of COX-2 activity than normoxic controls. (Figure 6), but the marked decrease by the combination was evident even in hypoxia.

### Drug combination downregulates EMT and cell migration in glioma cells

**Figure 7:**
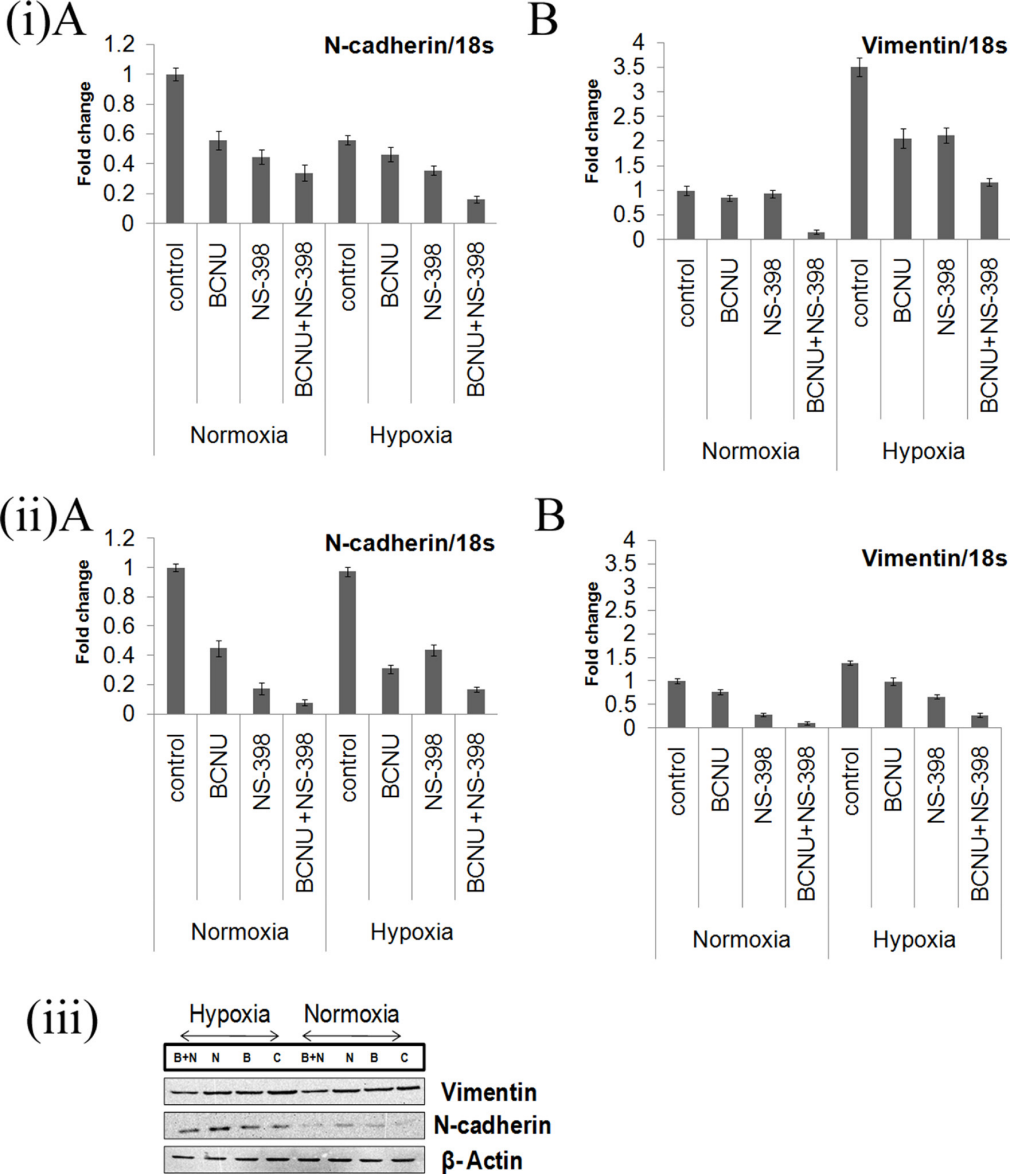
(**i**, **ii**) Real time PCR analysis of mRNA expression of EMT markers vimentin and N-cadherin. mRNA levels of the markers of epithelial to mesenchymal transition (Vimentin and N-cadherin) were more down-regulated by the combination of BCNU and NS-398 as compared to single agent in (i) LN229 and (ii) U87MG cell lines under hypoxia and normoxia. Results were expressed as mean ± SD of three experimental replicates. (**iii**) Western blot analysis of N-cadherin and Vimentin in the LN229 cell line. The protein levels of Vimentin and N-cadherin were also studied in the LN229 cell line. Here again, the decrease by the combination was more than that for single agent in both hypoxia and normoxia. Lane C denotes control, Lane B denotes BCNU, Lane N denotes NS-398 and Lane (B + N) denotes BCNU + NS-398.

**Figure 8:**
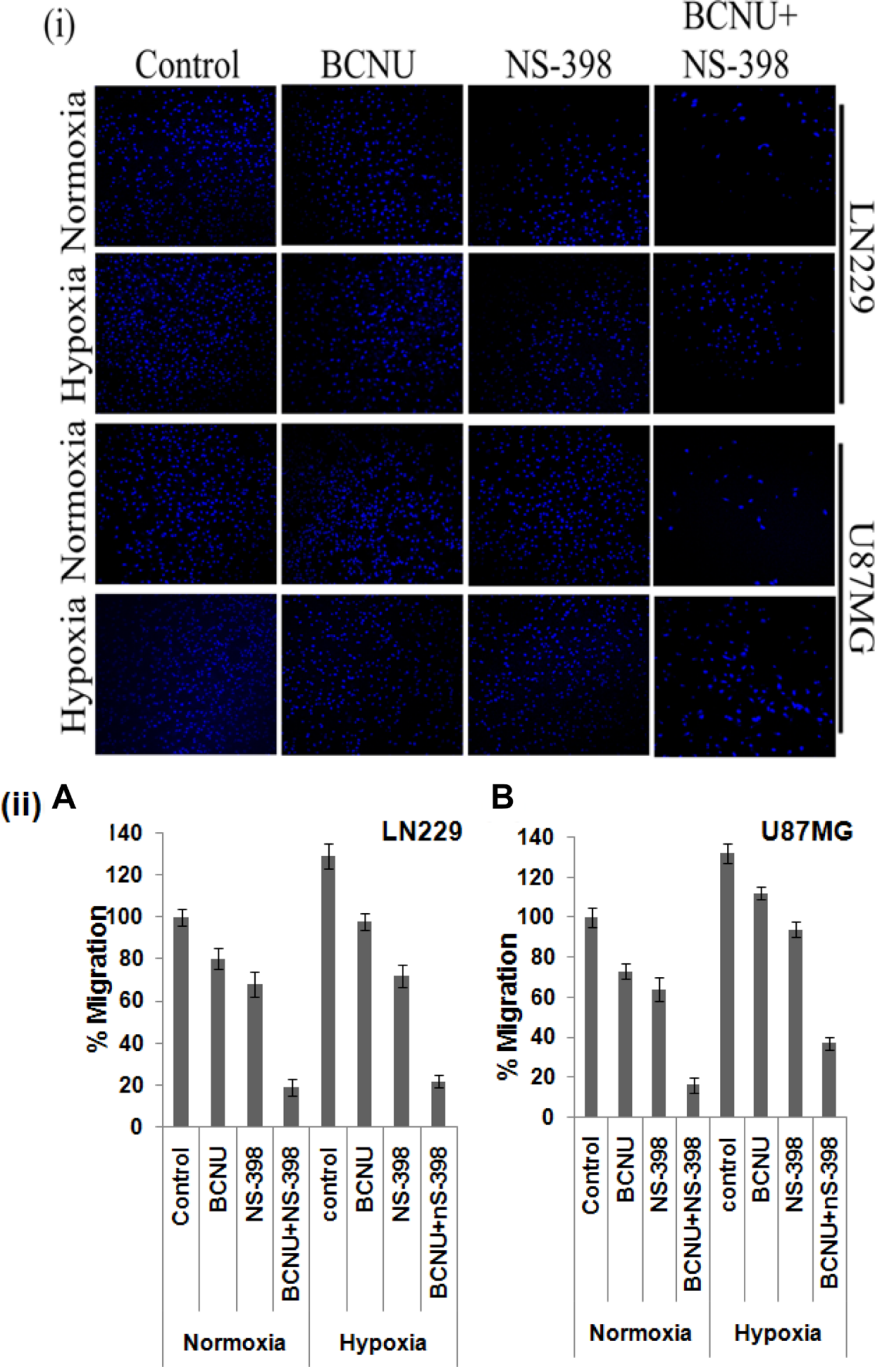
Cell migration as assessed by transwell membrane assay There was greater reduction in the migration of both cell lines after treatment with the combination as compared to the single agent under both hypoxia and normoxia. A pictorial representation of the cells is given in (**i**) while the percentage of the migrated cells are depicted in (**ii**). The combination had more effect than either single agent. This was true for normoxia (*p* < 0.01 with BCNU and *p* < 0.05 with NS-398) as well as hypoxia (*p* < 0.001 for BCNU and *p* < 0.01 for NS-398) for LN229. Also, for U87MG the levels of significance for normoxia were *p* < 0.01 for BCNU and *p* < 0.05 for NS-398. For hypoxia the levels of significance were *p* < 0.001 for BCNU and *p* < 0.01 for NS-398.

As COX-2 is reported to increase EMT, mRNA levels of EMT markers were evaluated individually and for the combination. Overall a greater decrease in the level of the EMT inducers, vimentin and N-cadherin, under hypoxia as well as normoxia was observed after treatment with the combination as compared to the individual drugs, indicating augmented reversal of EMT parameters (Figure 7(i, ii)). Decrease in the level of these EMT marker proteins by the combination as compared to single agent in LN229 cell line corroborated with mRNA level findings (Figure 7(iii)). EMT is directly correlated with migration that was studied by trans-well membrane assay. A greater reduction in the cell migration was seen by the combination as compared to the individual drugs (Figure 8(i)). There was significant reduction in the percentage of migration of the cells exposed to the combination as compared to the single agent (Figure 8(ii)).

### Efficacy of the drug combination on glioma stem cells

**Figure 9:**
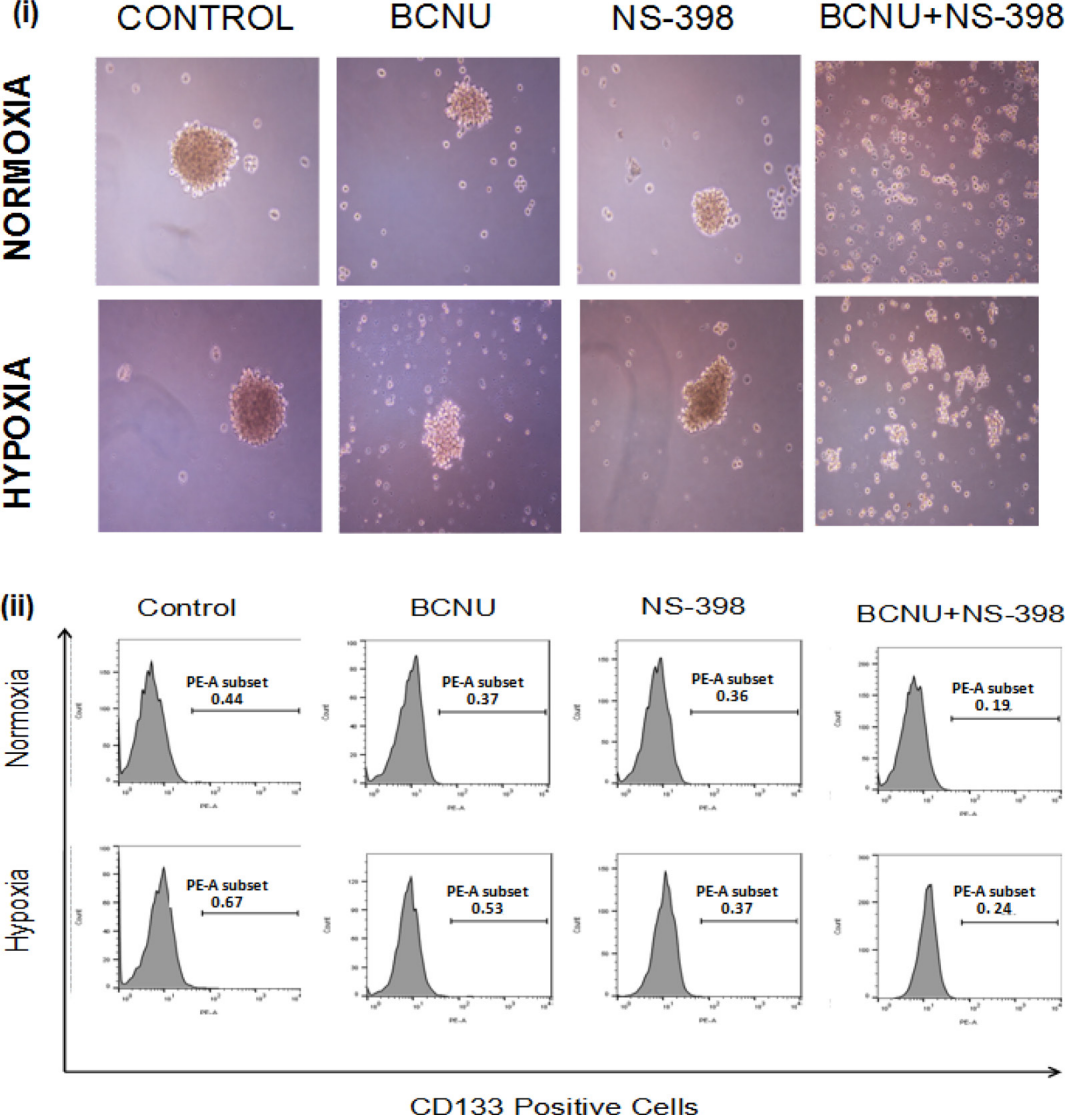
Photomicrographs of (**i**) gliomaspheres of U87MG cells. There was reduction in the size of the gliomasphere of the U87MG cells after treatment with the single agent under both hypoxia and normoxia. After treatment with the combination, the formation of gliomaspheres was abrogated in both the conditions, though some small cell clusters of less than 100 μm were observed. (**ii**) Analysis of CD133 expression in U87MG gliomaspheres. After 48 hours of drug treatment of gliomaspheres under hypoxia and normoxia, expression of CD133 was checked through flow cytometry. There was reduction in the expression of CD133 in gliomaspheres treated with the combination (BCNU + NS-398) as compared to the single agent under both hypoxia and normoxia. The number on each plot depicts the percentage of CD133 positive cells.

COX-2 is known to regulate the proliferation of cancer stem cells and its expression is aggravated by hypoxia. The effect of combination has been studied only in U87MG cells since LN229 do not form gliomaspheres under the usual growth conditions. We further found that treatment of the gliomasphere with the combination resulted in the abrogation of gliomasphere formation, though scattered clusters of cells were observed (Figure 9(i)). Abrogation of gliomasphere was indicated by reduced size as well as number of gliomaspheres (Table 1A and 1B). This was apparent in both hypoxia and normoxia. The experiment was continued for 10 days. There was no further growth of cells in the combination. CD133 is known to be glioma cancer stem cell marker, and was found to be upregulated under hypoxia as compared to normoxia. CD133 levels were reduced after drug treatment, and the effect of the drug combination was more than seen by for a single agent (Figure 9(ii)).

**Table 1A:** The mean size of gliomaspheres formed after drug treatment under normoxia and hypoxia in U87MG cell line

U87MG	Control	BCNU	NS-398	BCNU + NS-398
**Normoxia**	366.9 ± 80.54 μm	232.02 ± 68.21 μm	177.3 ± 53.9 μm	75.0 ± 17.01 μm
**Hypoxia**	359.42 ± 113.429 μm	158.48 ± 49.7 μm	210.13 ± 56.67 μm	71.73 ± 11.60 μm

It was seen that in the combination (Figure 9(i)), there was no compact sphere formation, but loose aggregrates are formed. The diameter of the same was measured. Diameter of aggregation of cells >100 μm was taken as sphere [48]. For U87MG gliomaspheres, the levels of significance for normoxia were *p* < 0.05 for BCNU and *p* < 0.01 for NS-398. For hypoxia the levels of significance were *p* < 0.01 for BCNU and *p* < 0.01 for NS-398.

**Table 1B:** The mean number of gliomaspheres formed after drug treatment under normoxia and hypoxia in U87MG cell line

U87MG	Control	BCNU	NS-398	BCNU + NS-398
**Normoxia**	112 ± 11	91 ± 7	86 ± 9	0
**Hypoxia**	72 ± 6	52 ± 9	58 ± 10	0

In the combination only poorly compacted aggregrates were seen.

## DISCUSSION

Grade IV glioma (Glioblastoma) show a high degree of hypoxia. Indeed hypoxia and neovascularization are amongst the essential criteria for histological diagnosis of glioblastoma. The hypoxic core of the tumor is responsible for many of the aggressive features of a tumor, including chemoresistance and reduced survival. Hypoxia inducible factors like HIFs are known to aggravate tumorigenesis by inducing epithelial to mesenchymal transition and stem cell like phenotype [38]. These cancer stem cell populations not only induce tumorigenesis but are also involved in reducing therapeutic efficacy of chemotherapeutic agents. Hypoxia is also known to maintain the self renewal capacity of cancer stem cells by the induction of stemness genes like Oct4, c-myc and Nanog [49, 50].

It has been reported that hypoxia regulates the expression of pro-inflammatory genes like COX-2, NOS2, PTX3 in glioblastoma cancer stem cells [51]. Up-regulation of COX-2 has been reported in many tumors and is implicated in increased resistance to cytotoxic therapies, cell proliferation, migration, invasion, angiogenesis and cancer stem like phenotype which helps in progression, metastasis and relapse of the malignancy [8, 52–54]. It has been established that Hypoxia Inducing Factor 1 alpha (HIF-1α), a regulator of oxygen homeostasis in tumor microenvironment, elevates COX-2 expression by regulating it at transcriptional level [55] and is also linked with increased levels of PGE2 [56]. Specific inhibitors of COX-2 have been shown to decrease cell proliferation upto a certain extent in many tumors including glioma. Many of such COX-2 inhibitors (Mavacoxib, Celecoxib) are in preclinical or clinical trials for attenuation of chemoresistance in gliomas or other cancers though they are still not commonly used [16, 17, 57–60].

Since progression of the disease depends on the interactions of several intinsic as well as extrinsic factors, recent therapeutic advancements focus on multi-modal drug combinations to target more than one potential molecule for better outcomes. The pathways being targeted by drug combinations include autophagy, PI3K/AKT pathway and angiogenesis. [6, 61–63]. This strategy can give rise to better therapeutic options for patient care. In this work we have tried to develop a strategy for glioma cells as well as cancer stem cells with high COX-2 expression, which would be effective in both hypoxia and normoxia. The broad aim is to deleteriously tackle the effects of protumorigenic inflammation.

We have earlier shown that COX-2 is over-expressed in a sub-set of GBM [64]. Our earlier work also shows that a member of the cadherin family, FAT1, drives COX-2 expression and is also a positive upstream regulator of HIF-1α, stemness and EMT under hypoxia [65, 66]. A prototype COX-2 inhibitor, NS-398 was used in this study. To further improve efficacy, NS-398 was combined with known anti-cancer drugs (BCNU, CP, 2-DG and TMZ) which affect different survival pathways of glioma cells. For comparative analysis of the cytotoxic effect, cells were also treated with either of the single agents under hypoxia and normoxia. For all drugs tested, there was relative resistance under hypoxia. BCNU was the only drug to show synergistic effect which overcame hypoxia induced chemoresistance. BCNU is recommended for the treatment of newly diagnosed high-grade glioma patients in whom 90% or more tumor has been resected. However, it does not appear to greatly prolong the medial survival [67–69]. It has been reported that as a single agent, BCNU is able to reduce the survival of the cancer cell but it is not able to kill the population of cancer stem cells [70]. For BCNU, there has been several clinical trials using it in combination with other drugs not only in glioma, but also in other cancers like melanoma, colorectal (https://www.clinicaltrials.gov/ct2/show/NCT00003346, NCT00005981). Some of the drugs used along with BCNU have been Temozolomide, O6-benzylguanine, acetaminophen, acetylcysteine (https://www.clinicaltrials.gov/ct2/show/NCT00362921, NCT00003346, NCT00005981, NCT00005637). For Celecoxib, the most commonly used COX-2 inhibitor, the combination trials have been mostly for other cancers like prostate, ovarian, colorectal (https://www.clinicaltrials.gov/ct2/show/NCT00215345, NCT01124435, NCT00230399). Some of the drugs used with celecoxib have included taxotere, carboplatin, capecitabine, Irinotecan (https://www.clinicaltrials.gov/ct2/show/NCT00215345, NCT01124435, NCT00230399). To our knowledge there has been no trial with a combination of BCNU and a COX-2 inhibitor. Since one of the drugs (BCNU) used in this study is already in clinical usage and other molecule (NS-398) is a prototype COX-2 inhibitor, the synergistic combination of these drugs may prompt the trials for this combination/equivalent drugs. Sub-lethal doses of the drug were used to simulate the intra-tumoral environment, where there is poor perfusion and reduced availability of the drugs in the interior core- a condition which induces drug resistance [71].

The combination of BCNU and NS-398 resulted in decreased cell viability and increased caspase dependent apoptosis of glioma cells under hypoxia as well as normoxia. There were concordant changes in pro and anti apoptotic markers. The effect of the combination went beyond merely additive to synergistic, under both hypoxia and normoxia. The synergistic action of the COX-2 inhibitor and BCNU was finally reflected in the reduction of PGE2, which was reduced more by the combination under both hypoxic and normoxic conditions. Even the enhanced levels of PGE2 in hypoxia showed greater reduction by the combination as compared to single agent. The effect was also seen on both EMT markers and cell migration.

It is important to note the effect of the combination on gliomaspheres, where the formation of distinct gliomaspheres was abrogated in both hypoxia and normoxia. Since a glioma stem cell like phenotype could be responsible for chemoresistance as well as recurrence of the malignancy, this combination has its potential to act through abrogation of stemness. CD133 which is a known glioma cancer stem cell marker [37] was also found to be reduced by the combination as compared to single agent.

To summarize, the unique feature of this combination that suppresses pro-tumorigenic inflammation is that it is effective under both hypoxia and normoxia. Inflammation is one of the pathways through which hypoxic chemoresistance acts, and the synergistic effects of this combination are evident at the level of both monolayer and gliomasphere cultures. As BCNU is already an approved drug and an increasing number of anti-inflammatory drugs notably COX-2 inhibitors are being approved for therapy, this prototype combination is worth investigating in further preclinical and clinical studies.

## MATERIALS AND METHODS

### Cell culture

Human glioma grade IV cell lines (LN229 and U87MG) were cultured as per ATCC recommendations. Cells were maintained under normoxic (20% O_2_, 5% CO_2_) and severe hypoxic conditions (0.2% O_2_, 5% CO_2_) in airtight chambers through Anoxomat gas proportionater (Mart Microbiology, Netherlands)

### Drug treatment and cell viability assay

Cells were seeded in 96 well plate and further they were exposed to hypoxia and normoxia. After further 24 hours, drug treatment was given from the respective stock solutions of CP, 2-DG, TMZ, BCNU and NS-398. MTT assay was done after 72 hrs of drug treatment as described by Jalota *et al*. 2016 [6].

### Combination index analysis

Drug interaction study was carried out based on combination index (CI) as described by Zhao *et al.* [47]. This clearly distinguishes additive effects from synergism, and a CI of less than 0.9 is taken as synergistic.

### Analysis by flow cytometry (PI and AnnexinV staining)

Cells were seeded and then synchronized for 8 hours. Further, they were exposed either to hypoxia or normoxia. After further 24 hours, drug treatment (BCNU, NS-398 and combination of both) was given. Study of percentage of apoptotic cells was done after 72 hours of drug treatment as described by Jalota *et al*. 2016 [6]. PI and Annexin V staining was done after 48 hours of drug treatment (BCNU, NS-398 and combination of both). Cells were trypsinized, washed and stained with PI and Annexin V. Acquisition of cells was done in BD FACSCanto. Analysis of dual staining was done by BD FACSdiva software.

### Immunoblotting

Cells were treated for 48 hours under hypoxia and normoxia and cell lysates were made and Western blotting was done as described by Jalota *et al*. 2016 [6]. COX-2, Bax, Vimentin and N-cadherin antibody were purchased from Cell Signaling. Bcl2 and β-actin antibody were procured from Abcam.

### RNA Isolation and real time PCR

RNA isolation and cDNA synthesis was done after drug treatment under hypoxia and normoxia as described by Jalota *et al*. 2016 [6]. To check the expression of the genes at mRNA level using cDNA, Real time PCR was done using gene specific primers and fluorescent SYTO9 dye. List of primers is mentioned in Supplementary Table 1.

### Caspase 3/7 assay

Cells were seeded in 96 well plate and after 24 hours transferred to different oxygen concentrations (20% O_2_ and 0.2% O_2_). Caspase 3/7 activity was determined using Caspase-Glo 3/7 assay (Promega) after 48 hours of drug treatment (BCNU, NS-398 and combination of both) according to manufacturer's protocol.

### PGE_2_ ELISA

Cells were seeded and after 24 hours transferred to different oxygen concentrations (20% O_2_ and 0.2% O_2_) and after further 24 hours drug treatment was given. Culture supernatants of the drug treated cells were collected after 72 hours of drug treatment. The concentration of PGE_2_ secreted into the medium was measured with an EIA kit for human PGE_2_ (Cayman Chemical) following manufacturer's instruction.

### Migration assay

Cells were seeded in 6 well plate and after 48 hours of drug treatment under hypoxia and normoxia, cells were proceeded using Boyden transwell chambers for migration assay according to manufacturer's protocol. The cells which migrated the transwell were counted after 24 hours of drug treatment for U87MG and LN229 cell lines. Number of cells which migrated the transwell were observed under microscope. Cells were counted in five different fields.

### Culture of gliomaspheres and anaylsis of size, number of spheres as well as stemness after drug treatment

Glioma cells U87MG were cultured in DMEM F-12 media (Gibco) supplemented with B27 (Gibco), bFGF and EGF supplement. Gliomasphere formation started at 3th day under both hypoxia and normoxia. After 5 days of gliomasphere formation under hypoxia and normoxia, drug treatment was given to gliomaspheres under hypoxia and normoxia. After further 24 hours, gliomaspheres were dissociated and cells were allowed to form gliomaspheres for 48 hours after drug treatment. Total number of spheres were then observed, counted and their diameters determined by the NIS elements BR 2.3. Only those compact aggregrates that had a diameter of more than 100 μm were taken as spheres [48]. For assessment of CD133 positive cells, with or without drug treatment, cells were allowed to form gliomaspheres and treated with drug or combination as described above. After 48 hours of drug treatment, cells were dissociated and stained for CD133. For staining, gliomaspheres were dissociated, washed and incubated with CD133 antibody (Miltenyi Biotech) in BSA at room temperature. Cells were then again washed and fixed with 4% paraformaldehyde. Cells fluorescence was acquired in BD FACS Canto. Analysis was done by Flow Jo Software.

### Cell viability after addition of pan caspase inhibitor

Drug treatment was given in combination with caspase inhibitor Q-VD-OPh (Calbiochem) to glioma cells (LN229 and U87MG) after further 24 hours of hypoxia and normoxia. Cells were treated in different groups with or without caspase inhibitor. These included either of the single agents as well as the combination. After 48 hours of treatment, caspase 3/7 assay was done and after 72 hours cell viability was done by MTT assay. Cell viability assay for the drug combination in comparison to control for cells which were treated with or without caspase inhibitor was done at 24, 48 and 72 hours after drug exposure.

### Statistical analysis

Statistical differences were assessed using a Student's *t*-test and a probability (*p*) value < 0.05 was considered significant. Results are expressed as mean ± SD of three experimental triplicates. All the experiments were done in triplicate and each experiment was repeated atleast three times. The results of the experiment presented in the figures are from the experimental triplicates.

## SUPPLEMENTARY MATERIALS FIGURES AND TABLES



## Abbreviations

GB: Glioblastoma
EMT: Epithelial Mesenchymal Transition
TMZ: Temozolomide
CP: Cisplatin; 2-Deoxy-D-glucose
CSC: Cancer stem cells
EGFR: Epithelial growth factor receptor
PGE_2_: prostaglandins E2
COX: 2- Cycloxoxygenase 2
HIF-1α: Hypoxia inducible Factor-1α.
