# Supplementary material for: A drug combination targeting hypoxia induced chemoresistance and stemness in glioma cells
Source: Oncotarget. 2018 Apr 6;9(26):18351–66. doi: 10.18632/oncotarget.24839 (PMC5915077; doi:10.18632/oncotarget.24839)
Supplement: Supplementary file 1 [file oncotarget-09-18351-s001.pdf]

A drug combination targeting hypoxia induced chemoresistance and stemness in glioma cells

SUPPLEMENTARY MATERIALS

REFERENCES

1. Irshad K, Mohapatra SK, Srivastava C, Garg H, Mishra S, Dikshit B, Sarkar C, Gupta D, Chandra PS, Chattopadhyay P, Sinha S, Chosdol K. A combined gene signature of hypoxia and notch pathway in human glioblastoma and its prognostic relevance. PLoS One. 2015; 10:e0118201. https://doi.org/10.1371/journal.pone.0118201.

2. Srivastava C, Irshad K, Dikshit B, Chattopadhyay P, Sarkar C, Gupta DK, Sinha S, Chosdol K. FAT1 modulates EMT and stemness genes expression in hypoxic glioblastoma. Int J Cancer. 2018; 142:805–12. https://doi.org/10.1002/ijc.31092.

Table 1 : List of primers

Target genes	Sequences		Annealing Temp.
18s rRNA	Forward<br>Reverse	GTAACCCGTTGAACCCCATT<br>CCATCCAATCGGTAGTAGCG	55–60° C
Bax	Forward<br>Reverse	CTTCAGGGTTTCATCCAG<br>CAGTTGAAGTTGCCGTCAGA	58° C
Caspase 3	Forward<br>Reverse	CTGCCTCTTCCCCCATTCT<br>CCAGAGTCCATTGATTCGCT	58° C
Cytochrome c	Forward<br>Reverse	CCAGTGCCACACCGTTGAA<br>TCCCCAGATGATGCCTTTGTT	58° C
Bcl2	Forward<br>Reverse	CTGCACCTGACGCCCTTCACC<br>CACATGACCCCACCGAACTCAAAGA	65° C
COX-2	Forward<br>Reverse	CTGCTCAACACCGGAATTTT<br>TTGAATCAGGAAGCTGCTTTT	58° C
Vimentin	Forward<br>Reverse	CAGCTAACCAACGACAAA<br>CGTGGAGTTTCTTCAAAAG	59° C
N-cadherin	Forward<br>Reverse	GCCACCTACAAAGGCAGAA<br>ATGTGCCCTCAAATGAAACC	59° C

Primers of hypoxia markers (CA9, VEGF and PGK1) and Vimentin were also used [1, 2].

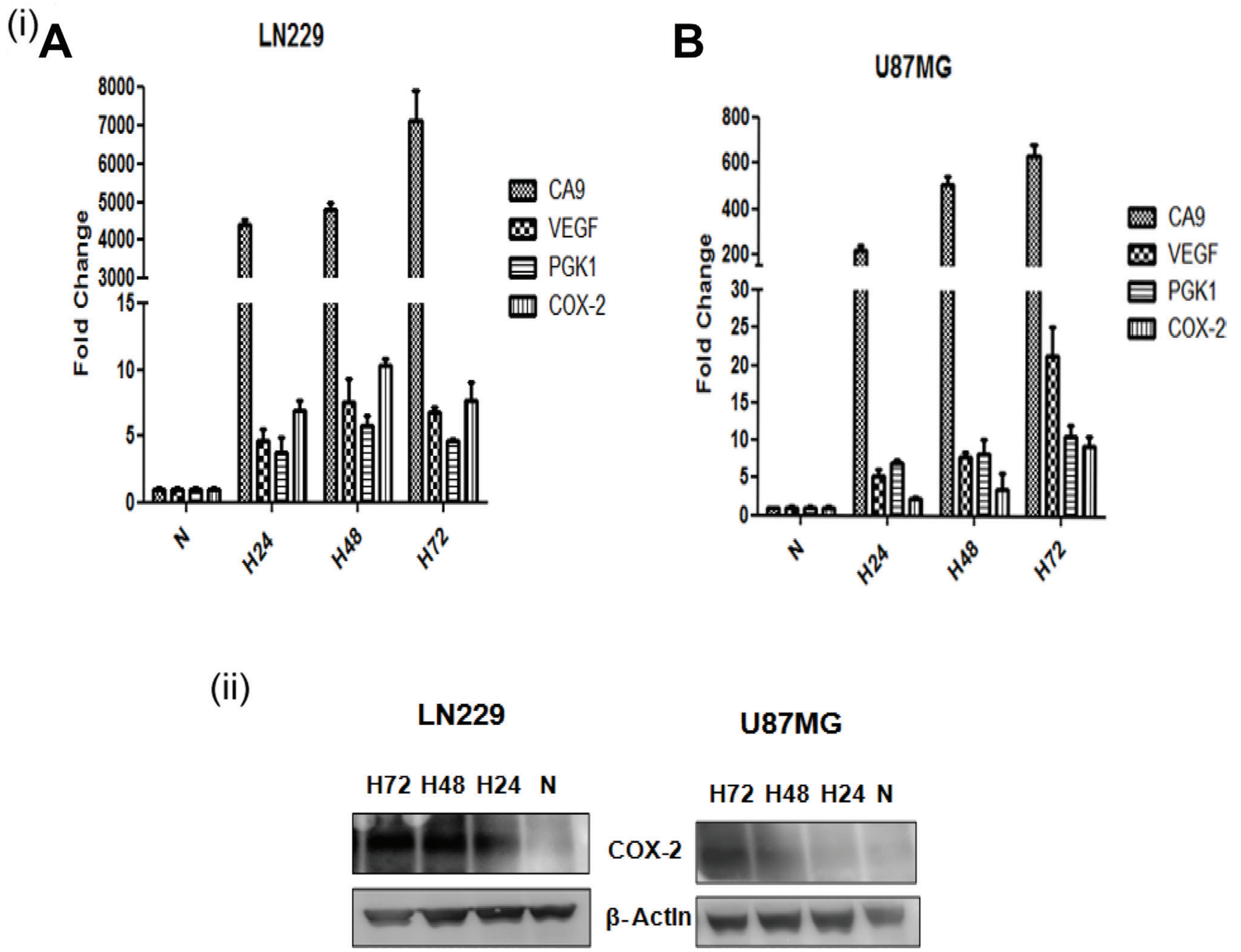

Supplementary Figure 1: (**i**) mRNA expression of Hypoxia markers (CA9, VEGF and PGK1) and COX-2 under normoxia and hypoxia in glioma cells. COX-2 mRNA expression was analysed using Real time PCR under 24, 48 and 72 hrs of hypoxia with respect to normoxia control in (A) LN229 and (B) U87MG cell lines. Upregulation of hypoxia markers and COX-2 expression was observed at all time points, being higher at 48 and 72 hours of hypoxia in LN229 and U87MG cell lines. (**ii**) Analysis of COX-2 protein expression in glioma cell lines. COX-2 protein expression was analysed under 24, 48 and 72 hrs of hypoxia with respect to normoxia control in LN229 and U87MG cell lines. Upregulation of COX-2 expression was observed at all time points, being maximum at 72 hours of hypoxia. N and H denotes Normoxia and Hypoxia respectively.

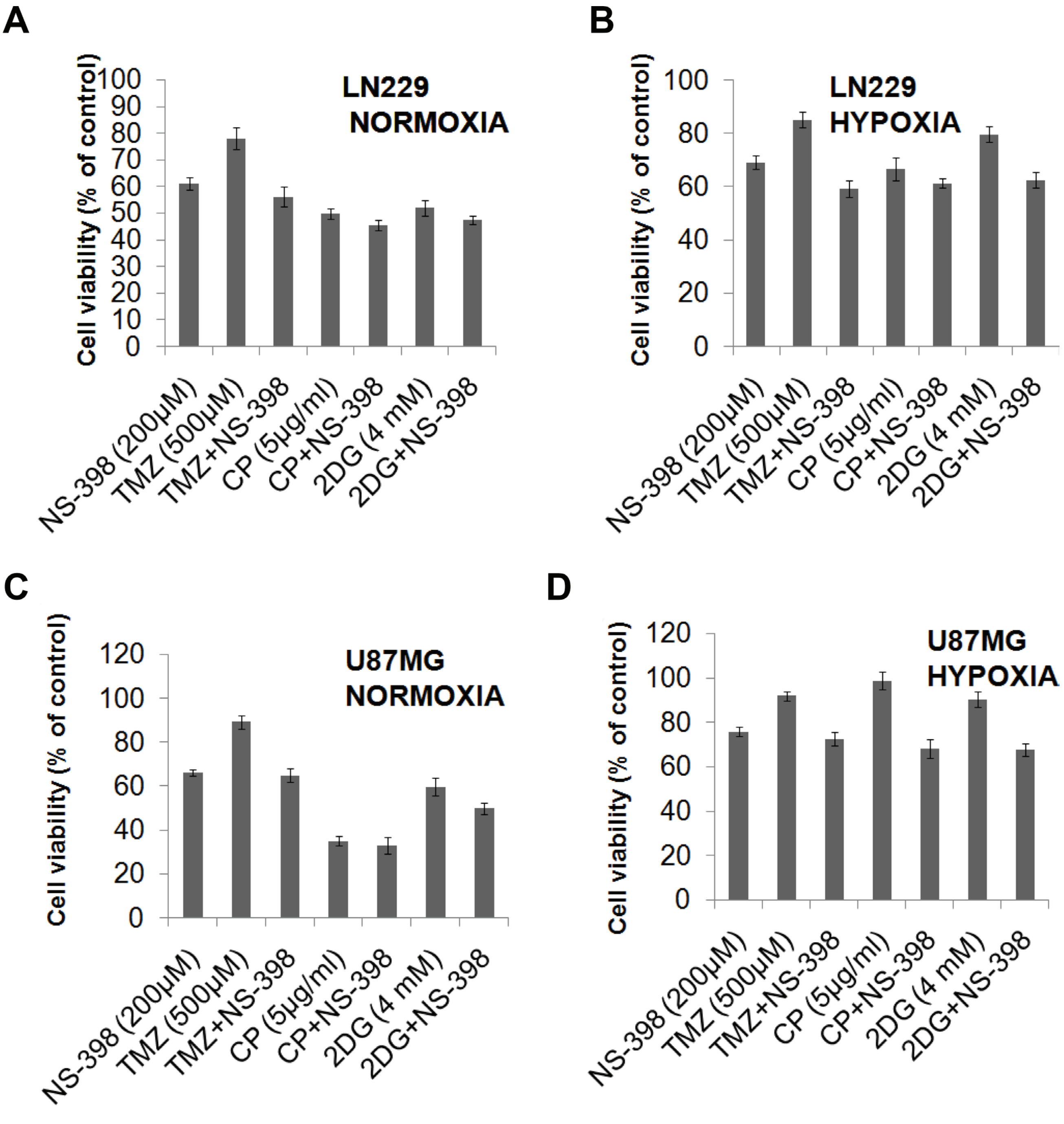

Supplementary Figure 2: Cell viability analysis of (**A**, **B**) LN229 and (**C**, **D**) U87MG cell lines after treatment with combination of NS-398 with CP, 2-DG and TMZ under normoxia and hypoxia. Cells were treated with NS-398 in combination with drugs (CP, 2-DG, TMZ) and cell viability was assessed by MTT assay after 72 hours. None of the combination showed any decrease in cell viability as compared to the single agent. Results were expressed as mean ± SD of three experimental replicates.

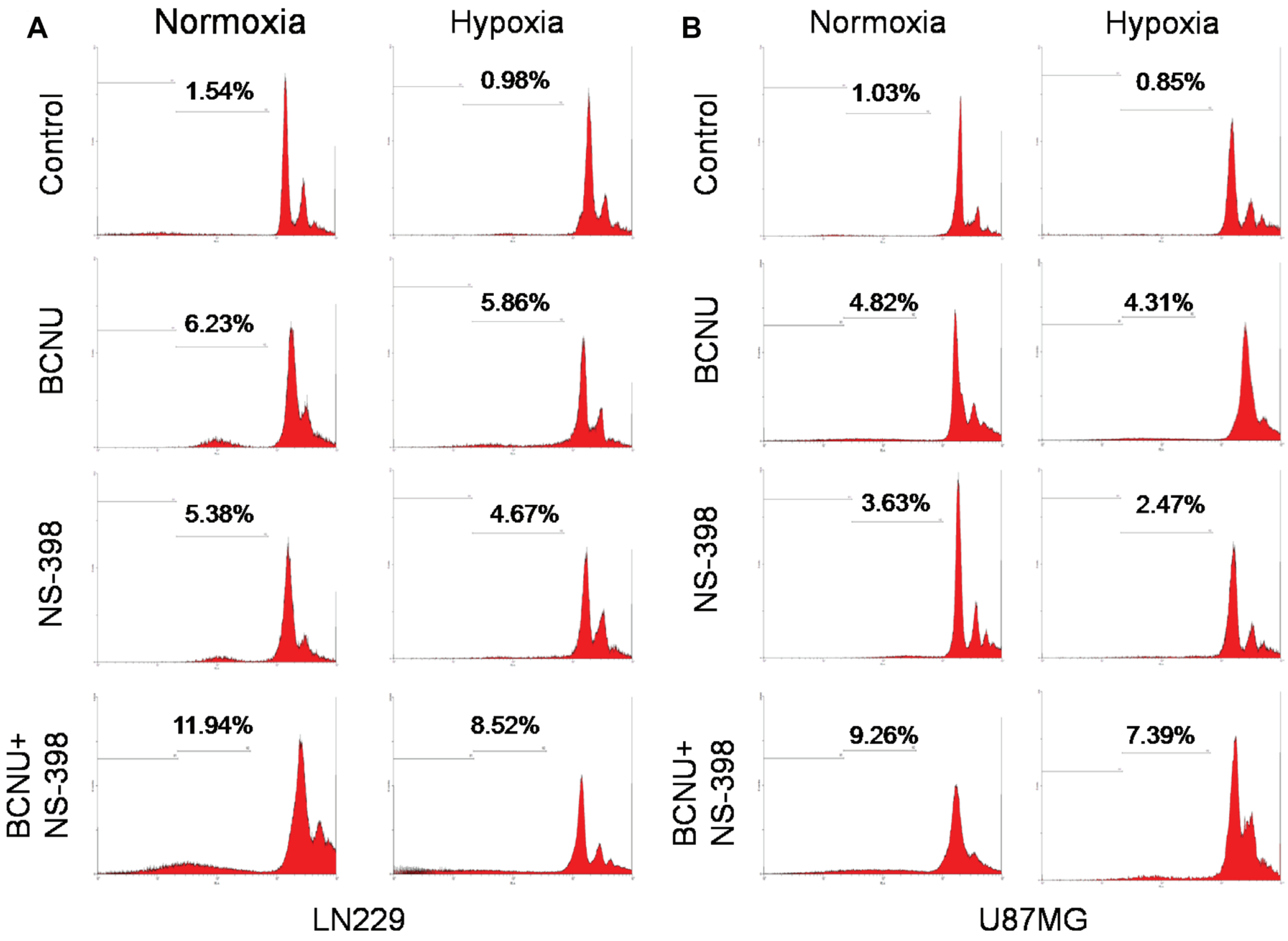

Supplementary Figure 3: Flow cytometric analysis by propidium iodide staining under various conditions. After 72 hours of drug treatment, cells (LN229 and U87MG) were fixed and stained with PI to determine the percentage of sub-G1 cells, indicating apoptosis. The combination of BCNU and NS-398 resulted in increased apoptosis (as indicated by the sub-G1 fraction), under both hypoxia and normoxia, as compared to single agent. (Panels **A** and **B**) are for LN229 and U87MG cell lines respectively.

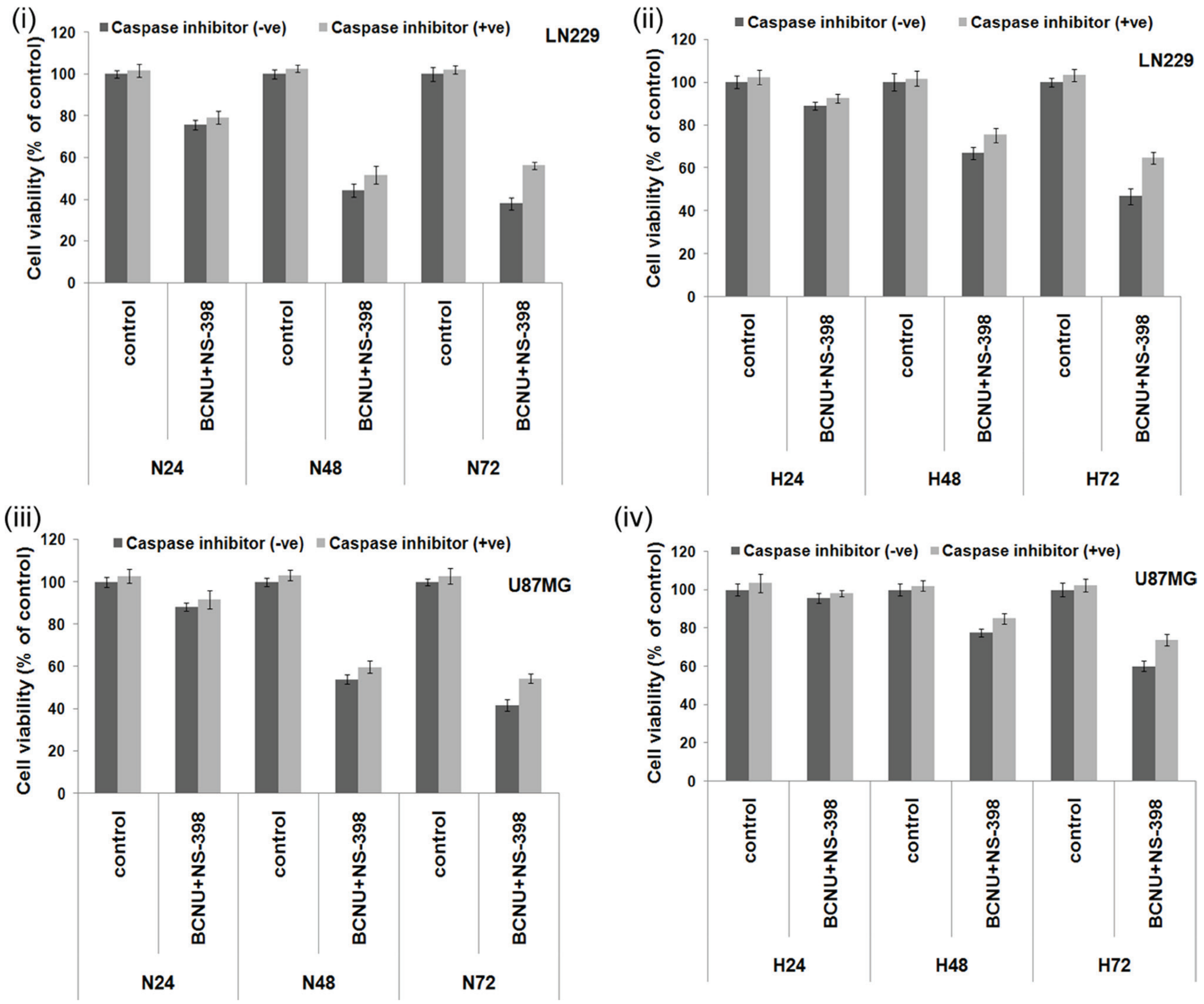

Supplementary Figure 4: Effect of the combination and caspase inhibitor on cell viability. Cells were treated with BCNU + NS-398 with or without caspase inhibitor and cell viability was assessed after 24, 48 and 72 hours in (**i**, **ii**) LN229 and (**iii**, **iv**) U87MG cell lines. The effect of the combination started at 48 hours and was more evident at 72 hours of treatment. Results were expressed as mean ± SD of three experimental replicates. N and H denotes Normoxia and Hypoxia respectively.
